# The ventromedial prefrontal cortex is particularly responsive to social evaluations requiring the use of person-knowledge

**DOI:** 10.1038/s41598-019-41544-z

**Published:** 2019-03-25

**Authors:** Tzipporah P. Dang, Bradley D. Mattan, Jennifer T. Kubota, Jasmin Cloutier

**Affiliations:** 1 Department of Psychological and Brain Sciences, University of Delaware, Newark, USA; 2 Department of Political Science and International Relations, University of Delaware, Newark, USA

## Abstract

Humans can rely on diverse sources of information to evaluate others, including knowledge (e.g., occupation, likes and dislikes, education, etc.) and perceptual cues (e.g., attractiveness, race, etc.). Previous research has identified brain regions supporting person evaluations, but are evaluations based on perceptual cues versus person-knowledge processed differently? Moreover, are neural responses consistent when person-knowledge is available but unnecessary for the evaluation? This fMRI study examined how the use and availability of person-knowledge shapes the neural underpinnings of social evaluations. Participants evaluated well-known actors based on attractiveness or body of work (i.e., person-knowledge) and unknown models based on attractiveness only. Analyses focused on the VMPFC, following research implicating this region in positive evaluations based on person-knowledge. The VMPFC was sensitive to the (1) availability of person-knowledge, showing greater responses as ratings became more positive for actors (but not models) regardless of rating dimension and (2) use of available person-knowledge, showing greater activity as ratings for likability based on body of work became more positive for actors versus models rated on attractiveness. These findings indicate that although brain regions supporting person evaluation are sensitive to the availability to person-knowledge, they are even more responsive when judgments require the use of available person-knowledge.

## Introduction

We can efficiently form impressions of others based on a multitude of perceptual characteristics. For example, our evaluations can be based on perceptual cues conveying social traits^1,2^ or group membership^3^. Although evaluations based on perceptual cues are efficient^4^ and ubiquitous^1,2^, the availability of person-knowledge (i.e., target-specific biographical information) allows more individualized evaluations^5^. Conceptually, these two sources of knowledge are thought to be processed simultaneously and interactively^6^.

Although the majority of social neuroscience research focuses on how perceptual cues alone affect evaluations^7–12^, a few studies have compared social judgments based on the simultaneous presentation or use of both perceptual cues and person-knowledge^13–19^. These studies have primarily examined perceived consistency of appearance-behavior pairings^13,15,17,18^ or the impact of perceptual and knowledge-based familiarity on face perception^14^. One transcranial magnetic stimulation study reported a causal role for the dorsomedial prefrontal cortex (DMPFC) in processing new information about faces previously paired with positive and negative person-knowledge^15^. In that study, participants formed impressions of congruent face-behavior pairs (e.g., a trustworthy face paired with sentences conveying positive behaviors) and then indicated whether their impressions were consistent with subsequently presented trait words. Researchers found that the DMPFC played a causal role in updating impressions based on new person-knowledge^15^. Other research suggests that such sensitivity to valenced person-knowledge in the DMPFC is relatively spontaneous^20,21^.

Importantly, theoretical and empirical work suggests that more ventral aspects of the medial prefrontal cortex (e.g., VMPFC) may more directly index increasingly positive explicit evaluations of others^9,22–25^ compared to the DMPFC. In the present study, we therefore examined activity in the VMPFC and other exploratory regions (e.g., DMPFC) as participants explicitly evaluated people based on (1) perceptual cues or person-knowledge when such knowledge was available (e.g., for known actors) or (2) perceptual cues only when person-knowledge was unavailable (e.g., for unknown models). A direct examination of percept- and knowledge-based forms of explicit evaluations, both independent of one another and in combination, is necessary to the development of a more comprehensive understanding of the neural processes supporting person evaluations. Although both kinds of evaluations may indeed recruit a similar set of brain regions, novel insights into person evaluation can be gained by identifying (1) the relative impact of percept- versus knowledge-based information on regions supporting explicit person evaluations, (2) the convergences and divergences in regions across this network (e.g., VMPFC vs. DMPFC), and (3) the combined impact when both percept- and knowledge-based information are present.

## Perceptual Cues

Perceptual cues provide valuable information that can be gleaned just from looking at another person. For example, we can often gather information about someone’s socioeconomic status via clothing, dominance via physical size, or trustworthiness via facial expression^12,26^. Many neuroimaging studies on person evaluation have focused on novel faces lacking prior knowledge associations^20,27,28^. Irrespective of accuracy, perceptual cues frequently guide evaluations as they are often the only or most salient source of social information^29^.

Past research has implicated different neural regions in various percept-based evaluations. For example, the amygdala is frequently implicated in studies involving race^9,10,30^, valence of emotional expressions^29,31^, or trustworthiness based on facial cues^26^. Some regions have been shown to respond to variations in attractiveness. For example, the ventral striatum has been shown to be involved in reward processing and positive attractiveness ratings^32,33^. To a lesser extent than the amygdala and ventral striatum, the VMPFC has also been shown to be sensitive to positive evaluations of others (e.g., positive ratings of facial attractiveness)^29^.

## Person-knowledge

Although evaluations based solely on perceptual cues (e.g., attractiveness) are efficient, we also evaluate others based on person-knowledge^27,28^, often leading to more complex individuated impressions^5^. In the present study, we examined brain regions associated with the availability and use of person-knowledge during explicit evaluations. Specifically, we were interested in whether these processes are supported more by the DMPFC or VMPFC. The DMPFC has been implicated in various aspects of thinking about people including the retrieval of person-knowledge^14,20,34,35^, the formation and updating of impressions^15,16,36,37^, and mentalizing^38–40^. The VMPFC is also believed to support relatively explicit social evaluations based on person-knowledge, with typically greater activity for positively evaluated people^12,23–25,41,42^. Consistent with its sensitivity to both social and evaluative information^43^, meta-analyses and reviews of the literature purport that the VMPFC more broadly supports the flexible generation of affective meaning by integrating evaluation-relevant information from multiple dimensions and time points^25,29,44,45^. In other words, the VMPFC is suggested to support the process of combining current environmental cues and past knowledge, possibly to predict future outcomes. In the context of person evaluation, the VMPFC is sensitive to explicit positive evaluations based on previous knowledge^12,23,41^. For example, greater VMPFC activity is observed when person-knowledge about someone’s high compared to low moral status is available^23,41^. Moreover, the VMPFC is sensitive to personally relevant and well-liked individuals about whom perceivers have extensive knowledge^46–49^. Based on previous research implicating the VMPFC in explicit evaluations solely from perceptual cues^29^ or solely from person-knowledge^23,41^, we focused on this region as our primary region of interest (ROI). We anticipated that the VMPFC may be particularly involved in person evaluations based on the simultaneous presentation of both types of information.

## Study overview

In the current event-related fMRI investigation, we examined how the availability and use of person-knowledge shapes neural responses during percept- and knowledge-based evaluations. Specifically, participants evaluated: (1) unknown models on attractiveness (i.e., absence of person-knowledge), (2) well-known actors on attractiveness (i.e., availability of person-knowledge that is irrelevant to evaluations), and (3) well-known actors on likability based on body of work (i.e., availability of person-knowledge is required for evaluations). We focus on attractiveness and body of work because these two dimensions best allow us to differentiate the influence of perceptual cues and person-knowledge. Attractiveness judgments can be made based solely on perceptual cues and in the absence of person-knowledge (e.g., unfamiliar models). However, even if person-knowledge is available, one can presumably assess attractiveness based on perceptual cues without being influenced by person-knowledge (e.g., familiar actors). On the other hand, body-of-work judgments rely on person-knowledge and should not depend on attractiveness. We first examined the relative impact of the absence, mere availability, and use of person-knowledge during deliberative evaluations by identifying potential increases in VMPFC activity in response to increasingly positive physical-based evaluations of models (i.e., attractiveness), increasingly positive physical-based evaluations of actors (i.e., attractiveness), and increasingly positive knowledge-based evaluations of actors (i.e., likability based on body of work), respectively. We additionally compared each of the three conditions to each other to determine whether the relationship between positive ratings and VMPFC activity was especially pronounced for a given form of person evaluation.

### Predictions

Based on previous research^29^, we anticipated that the VMPFC would generally respond to targets associated with relatively positive (vs. negative) evaluations regardless of rating dimension (i.e., body of work vs. attractiveness). Additionally, the VMPFC may be sensitive to positive evaluations when person-knowledge is available (vs. not available). Specifically, we predicted preferential activity following positive evaluations when person-knowledge is either available (viz., evaluating the physical attractiveness or body of work for well-known actors) or required during person evaluation (viz., evaluating actor likeability vis-a-vis their body of work). In other words, we expected the VMPFC to be more involved when evaluating actors more positively regardless of rating dimension compared to when evaluating models more positively based on attractiveness. Lastly, the VMPFC may be particularly sensitive to positive evaluations when person-knowledge is required. Specifically, we predicted preferential recruitment of the VMPFC when evaluating actors based on person-knowledge (i.e., likeability vis-à-vis their body of work) versus perceptual cues (i.e., attractiveness).

Because it has also been implicated in the relatively more spontaneous retrieval of person-knowledge^20,21,34,35^, we also explored neural responses to the availability and use of person-knowledge in the DMPFC. We anticipated that this region may be particularly sensitive to the availability of person-knowledge when it is not explicitly required for evaluations.

## Results

### ROI analyses

Our primary analyses focused on the VMPFC as our a priori ROI. We conducted exploratory analyses for other regions relevant to person evaluation, including the DMPFC. Because all effects in the DMPFC were non-significant, we report only results from the VMPFC here. (See Supplementary Material S2 for full results from all exploratory ROIs.)

#### Do evaluations based on person-knowledge or attractiveness modulate VMPFC activity?

We first examined whether increased positive evaluations led to greater VMPFC activity when those evaluations were based on (1) perceptual cues without person-knowledge (i.e., models rated on attractiveness), (2) perceptual cues with available person-knowledge (i.e., actors rated on attractiveness), and (3) person-knowledge (i.e., actors rated on body of work). Ratings provided in the scanner were included as parametric predictors separately for each of the three conditions in our design (see Materials and Methods). We then conducted separate one-sample t-tests on parameter estimates for each of the three parametric predictors (i.e., models rated on attractiveness, actors rated on attractiveness, and actors rated on likability based on body of work) compared to zero. This analysis was conducted to examine the potential involvement of the VMPFC in supporting positive ratings within each condition separately.

We observed a significant VMPFC involvement for evaluations based on perceptual cues with available person-knowledge (i.e., actors rated on attractiveness), *t*(54) = 2.672, *p* = 0.010, such that VMPFC activity increased as ratings of attractiveness for actors became more positive. We also observed significant VMPFC involvement for evaluations based on person-knowledge (i.e., actors rated on likability based on body of work), *t*(54) = 3.660, *p* < 0.001, such that VMPFC activity increased as ratings of likability based on body of work became more positive. However, we did not observe a significant VMPFC involvement for percept-based evaluations made in the absence of person-knowledge (viz., models rated on attractiveness), *t*(54) = 0.678, *p* = 0.501. Taken together, these results indicate that the VMPFC is sensitive to positive evaluations when person-knowledge is available, irrespective of whether that person-knowledge is directly relevant to evaluations.

#### Do person evaluations modulate VMPFC activity more when person-knowledge is used or when it’s simply available?

To examine whether sensitivity to positive ratings was especially pronounced for a particular condition (e.g., use of person-knowledge: actors’ body of work) relative to other conditions (e.g., mere presence of person-knowledge: actors’ attractiveness), we next conducted a one-way ANOVA on parameter estimates to detect the presence of any differences amongst the parametric predictors.

**Figure 1:**
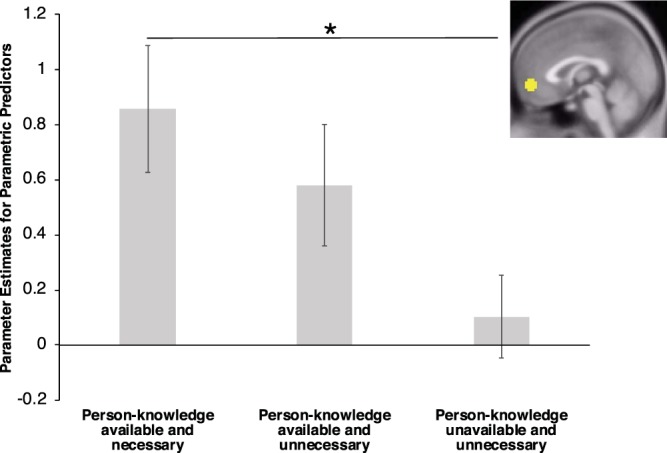
Magnitude of parametric modulation of VMPFC activity by positive ratings, plotted by condition. We conducted pair-wise contrasts of VMPFC parameter estimates for the parametric predictors when participants were rating actors on likability based on body of work (i.e., person-knowledge available and necessary), actors on attractiveness (i.e., person-knowledge available and unnecessary), and models on attractiveness (i.e., person-knowledge unavailable and unnecessary). Results indicate that VMPFC activity is particularly sensitive to the use of available person-knowledge relative to the absence of person-knowledge. The significant simple effect (*) is indicated, *p* = 0.010. All other pair-wise contrasts were non-significant, *p* > 0.104.

The results indicated that the parametric predictors differed in magnitude: significant effect of parametric predictor on VMPFC activity, *F*(2,162) = 3.479, *p* = 0.033. We followed up on this finding by conducting linear regressions to test all possible pair-wise contrasts between the parametric predictors. We found evidence of a significant linear relationship between positive ratings and the combined availability/use of person-knowledge (contrast codes: parametric predictor for actors’ body of work = 0.5, parametric predictor for actors’ attractiveness = 0, and parametric predictor for models’ attractiveness = −0.5), *b* = 0.753, *SE* = 0.288, *CI*_95%_ = [0.188, 1.317], *F*(1,163) = 6.833, *p* = 0.010. Specifically, VMPFC activity increased more as ratings of likability based on body of work for actors became more positive compared to when ratings of attractiveness for models became more positive. No other significant differences emerged: (1) ratings of likability based on body of work for actors compared to ratings of attractiveness for actors, *b* = 0.276, *SE* = 0.293, *CI*_95%_ = [−0.298, 0.851], *F*(1,163) = 0.889, *p* = 0.347; and (2) ratings of attractiveness for actors compared to ratings of attractiveness for models, *b* = 0.477, *SE* = 0.292, *CI*_95%_ = [−0.095, 1.048], *F*(1,163) = 2.670, *p* = 0.104. Taken together, these results indicate that the VMPFC is particularly sensitive to the use of available person-knowledge relative to the absence of person-knowledge (Fig. 1).

### Exploratory whole-brain analyses

Complementing our a priori focus on the VMPFC, we conducted exploratory whole-brain analyses to determine whether (1) any other regions (e.g., DMPFC) would show a similar pattern of activity to that observed in the VMPFC ROI and (2) the activity observed in our a priori ROI analysis would be robust to multiple comparison correction at the whole-brain level.

#### Analysis parameters

Separate whole-brain analyses at the second level were performed to examine increases and decreases in neural activity as a function of in-scanner ratings provided during each of the three conditions: (1) actors rated on body of work, (2) actors rated on attractiveness, and (3) models rated on attractiveness. Using the Monte Carlo simulations included in AlphaSim, the minimum cluster size required for a whole-brain correction at *p* < 0.05 with an uncorrected threshold of *p* < 0.001 was estimated to be 51 contiguous voxels. We summarize results from each of the whole-brain analyses below. The results for all analyses are reported in Table 1.

**Table 1:** Identification of BOLD signal as a function of Rating Dimension and Person-Knowledge.

Brain Region	*k*	*t*	*p*	MNI Coordinates		
				*x*	*y*	*z*
**Increasing with increasing body-of-work likability ratings for familiar targets (i.e., actors)**						
Calcarine Sulcus	95	5.01	<0.001	−3	−60	12
VMPFC	223	4.55	<0.001	6	63	0
L Superior Occipital Gyrus	57	3.99	<0.001	−27	−81	42
**Increasing with decreasing body-of-work likability ratings for familiar targets (i.e., actors)**						
N/A						
**Increasing with increasing attractiveness ratings for familiar targets (i.e., actors)**						
R Lingual Gyrus	76	4.09	<0.001	36	−75	6
**Increasing with decreasing attractiveness ratings for familiar targets (i.e., actors)**						
N/A						
**Increasing with increasing attractiveness ratings for unfamiliar targets (i.e., models)**						
N/A						
**Increasing with decreasing attractiveness ratings for unfamiliar targets (i.e., models)**						
R Superior Parietal Gyrus	112	4.55	<0.001	12	−81	51

*Note*. Exploratory whole-brain analysis of 55 participants (threshold = *p* < 0.001, uncorrected; clusters ≥51 voxels determined by AlphaSim; actual values are reported in the table).

#### Impact of person-knowledge use (actors rated on body of work only)

**Figure 2:**
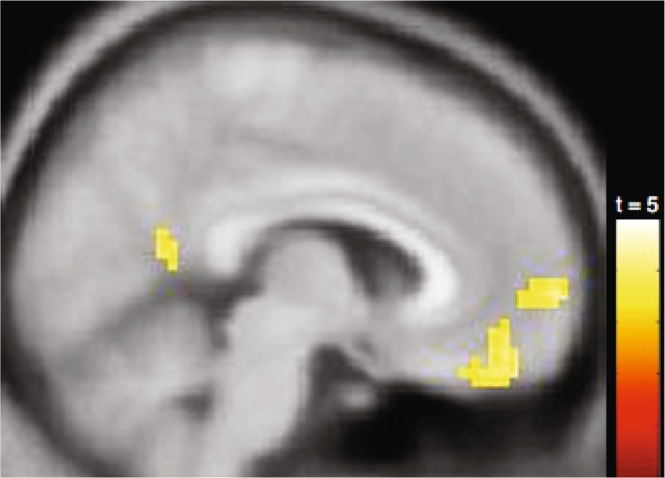
Brain regions associated with increases in body-of-work likability ratings for actors. The results of this exploratory whole-brain analysis are displayed on a sagittal section, x = 6 mm. Increased body-of-work ratings for the actors were associated with increasing activity in the VMPFC (peak MNI_x, y, z_ = [6, 63, 0]).

We observed greater activity in the calcarine sulcus, VMPFC, and superior occipital cortex as body-of-work ratings increased for the actors, but no reliable changes as body-of-work ratings decreased (Fig. 2). These findings converge with the ROI findings reported above showing that VMPFC activity was sensitive to increasing positivity in body-of-work ratings for well-known actors.

#### Impact of person-knowledge availability (actors rated on attractiveness only)

We observed greater activity in the lingual gyrus as attractiveness ratings increased for the actors, but no reliable changes as attractiveness ratings decreased. Although the VMPFC was reliably modulated by increasingly positive ratings of actor attractiveness in our reported ROI analyses, this region did not survive thresholds for multiple comparison in this corresponding whole-brain analysis.

#### Impact of percept-based evaluations without person-knowledge (models rated on attractiveness only)

We observed greater activity in the superior parietal gyrus as attractiveness ratings decreased for the actors, but no reliable changes as attractiveness ratings increased.

### Supplementary Analyses Controlling for Post-scan Stimulus Familiarity

In order to see if differences in perceptual familiarity (and not differences in person-knowledge, as hypothesized) could account for differences arising between comparisons of actors and models rated on attractiveness, we conducted analyses while controlling for post-scan familiarity ratings in Supplementary Material S2. Specifically, we re-analyzed the ROI data while accounting for post-scan familiarity ratings as a parametric modulator in the level-1 GLM. Results from these analyses were consistent with the corresponding original ROI analyses reported in the main text and Supplementary Material S2, suggesting that perceptual familiarity is unlikely to account for these differences.

Finally, in order to account for post-scan ratings of familiarity in the whole-brain exploratory analyses reported in the preceding section, we also re-analyzed these data while accounting for post-scan familiarity ratings as an additional parametric modulator in the level-1 GLM for each of the three whole-brain parametric analyses (see Supplementary Material S2). With the exception of clusters in the parietal cortex (see Supplementary Material S2), results from the whole brain analyses were consistent with the whole-brain findings reported in the preceding section (cf. Table 1 and Fig. 2).

### General Discussion

The goal of the current study was to examine how VMPFC activity is impacted by the availability and use of person-knowledge. Our findings indicate that the VMPFC is especially sensitive to the use of available person-knowledge but also to a lesser extent to the availability of person-knowledge. Moreover, person-knowledge may be spontaneously used in evaluations of others even when such knowledge is not necessary for the task at hand, such as when judging familiar people based on their attractiveness. Although existing research has implicated the VMPFC in explicitly assessed positive evaluations based on person-knowledge and, to a lesser extent, based on perceptual cues, these two forms of explicit evaluation have not been directly compared in a single brain imaging study.

Importantly, our predictions for the involvement of the VMPFC in positive explicit evaluations when person-knowledge was available were confirmed. VMPFC was more involved in increasingly positive evaluations when person-knowledge was required compared to when person-knowledge was unavailable. Although VMPFC activity also tracked with increasingly positive evaluations when person-knowledge was available but not necessary for evaluation, the magnitude of this relationship did not differ relative to the non-significant relationship between VMPFC activity and positive evaluations in the absence of person-knowledge. In contrast, our prediction that the VMPFC would be involved in positively evaluating others regardless of person-knowledge availability was not supported. It appears that the VMPFC may be less involved in evaluations of others based solely on perceptual cues in the absence of person-knowledge.

There are several key take-aways from these findings. First, results suggest that when person-knowledge is available it influences how we evaluate others, even when the knowledge in question is not necessary for the judgment at hand. Second, VMPFC may be particularly involved in the implementation of person evaluations requiring the use of person-knowledge. Third, the current findings suggest that contributions from the VMPFC to explicit evaluations critically depend on the presence of person-knowledge. In other words, this region does not appear to support positive evaluations based on a perceptual attribute in the absence of person-knowledge. Finally, these effects were observed in the VMPFC, but not the DMPFC (see Supplementary Material S2), illustrating an important divergence in the medial prefrontal cortex during explicit social evaluations, at least when person-knowledge is available.

Consistent with previous research, the VMPFC is not always sensitive to attractiveness in the absence of person-knowledge^50^. Indeed, with respect to attractiveness ratings, the VMPFC only showed enhanced sensitivity to ratings of attractiveness when person-knowledge about the targets was available (i.e., actors). These results provide further evidence of an extended neural system supporting the various aspects of person perception^28,34,35^. In particular, the VMPFC may be spontaneously sensitive to the availability of person-knowledge, even when that person-knowledge is not needed (e.g., when evaluating the attractiveness of familiar actors).

The VMPFC’s spontaneous sensitivity to person-knowledge during social evaluations is noteworthy given evidence that the DMPFC may also be involved in the spontaneous use of person-knowledge. However, one key difference is that the present study involved explicit evaluations. With few exceptions^16,37^, studies finding sensitivity in the DMPFC to valenced person-knowledge have tended to rely on indirect comparisons. At least during the critical contrasts reported in these studies, person-knowledge was not used for direct and explicit evaluations (e.g., impression consistency judgments^15^, n-back task^20,21,51^). Even when critical analyses involve explicit evaluations, the DMPFC is not directly sensitive to increasingly positive evaluations of others. Instead, the DMPFC appears to be sensitive to evaluative inconsistency^17,18^ or impression updating based on evaluatively inconsistent information^16,37^. For studies reporting DMPFC sensitivity to impression updating as a function of the valence of new information, the DMPFC is either not sensitive to valence^37^ or shows increased activity to increasingly *negative* impression updating^16^. This contrasts with the present finding that activity in the VMPFC (but not the DMPFC) tracks increasingly *positive* evaluations based on person-knowledge. Rather than directly indexing the valence of explicit evaluations based on person-knowledge as in the VMPFC, we speculate that the DMPFC is sensitive to potentially diagnostic person-knowledge that conflicts with an existing impression. Because participants in this study were not systematically presented with evaluatively inconsistent information (e.g., unattractive actors whose body of work is nonetheless impressive), it is perhaps to be expected that we did not observe activity in the DMPFC as a function of explicit evaluations or otherwise.

The present study also raises the question of whether the VMPFC is involved in the integration of the evaluative impact of perceptual and knowledge-based cues on social evaluations even when one or more of these cues are irrelevant to the explicit evaluation at hand. To better understand whether perceptual and knowledge-based information are integrated (vs. processed in parallel) in the VMPFC, future work will need to examine these processes in a more orthogonal fashion. For example, evaluating actors’ likability based on their body of work in the absence of perceptual cues (e.g., using names rather than faces) would provide a useful contrast with the same rating in the presence of both facial cues and person-knowledge. Greater VMPFC activity when rating actors’ body of work after seeing their faces compared with their names would provide clearer support that the VMPFC is involved in the integration of the evaluative impact from perceptual cues and person-knowledge.

Complementing our ROI approach, the whole-brain analyses provided convergent information about the role played by the VMPFC in person evaluation. Consistent with our ROI analyses of the VMPFC, we observed greater activity as body-of-work ratings increased for the actors. As mentioned previously, this pattern did not manifest in the DMPFC. However, we did observe effects in bilateral amygdala that were similar to those observed in the VMPFC ROI (see Supplementary Material S2).

Contrasting with the observed effects of person-knowledge in this study, we observed few effects of increasingly positive evaluations in percept-based evaluations (i.e., attractiveness judgments) in the whole-brain (see Results) or exploratory ROI analyses (see Supplementary Material S2). Regions emerging in analyses of attractiveness ratings (actors or models) did not coincide with areas previously implicated in reward or attractiveness (e.g., ventral striatum)^32,33^. It is possible that variability in another trait that is frequently conveyed through both perceptual and knowledge-based antecedents (e.g., competence^16^) may better delineate networks implicated in evaluations based on perceptual (vs. knowledge-based) cues.

In the current study, we used actors with whom participants were already familiar and therefore were not able to experimentally control for exact amount of information known about the actors. Nonetheless, we did attempt to control for effects of familiarity in our models in a set of supplemental analyses (see Supplementary Material S2). Results from these supplementary analyses were consistent with those reported in the main text, suggesting that perceptual familiarity is unlikely to account for observed differences, particularly those arising in the contrast of actors versus models for attractiveness ratings. Future research should explore how the amount and kind of knowledge impacts neural networks involved in person evaluation. Furthermore, it is worth nothing that there may not have been as much variability in the negativity of the targets utilized in the current study (in either attractiveness or body-of-work judgments; see Supplementary Material S1). As such, it would be useful in future research to provide participants with varying amounts of person-knowledge about novel individuals that equally varies in positivity and negativity. This would allow for a more systematic examination of the relative impact of valence and amount of knowledge on networks supporting person evaluation. Additionally, as our sample only included male participants, the current findings may not generalize to female participants because evaluations of attractiveness may be sensitive to perceiver gender^50^. However, it remains unclear how perceiver gender might affect evaluations based on person-knowledge.

In conclusion, our findings indicate that activity in the VMPFC is sensitive to positive evaluations when person-knowledge is available, irrespective of whether that knowledge is relevant to one’s evaluation. Previous research has found that key components of the extended brain networks supporting person perception are especially sensitive to faces associated with person-knowledge^17,20,21,52^. It will be critical for future research to examine the role of the VMPFC within this broader neural network, and particularly the degree to which this region may support the dynamic integration of perceptual and knowledge-based information during person evaluation and impression formation^6^.

## Materials and Methods

### Participants

Sixty-one self-identified White male participants were recruited from the Chicago area via leaflets, online postings, and advertisements on public transportation for monetary compensation ($60–105). See Supplementary Material S1 for screening procedures. Six participants who completed the study were excluded due to an incomplete post-scan familiarity questionnaire (*n* = 1), in-scanner ratings not being recorded (*n* = 1), excessive movement in the scanner (*n* = 1), and familiarity with the model stimuli and/or lack of familiarity with actor stimuli (*n* = 3; see Procedures and Supplementary Material S1). The remaining 55 participants were 18–35 years old (*M*_*age*_ = 24.255 years, *SD*_*age*_ = 4.608 years) and had normal or corrected-to-normal vision. Although this final sample size was approximately twice the size of an average fMRI study, it nonetheless falls short of recommended sample sizes for whole-brain across-participant analyses that adequately account for multiple comparisons^53^ (*n* > 80). Following the recommendation of Vul and Pashler^53^, we therefore focus our analyses on an a priori ROI. Previous analyses indicate that sample sizes needed for adequate power can be reduced by three to four times by adopting an ROI-based approach^54^. Participants provided informed consent in accordance with the experimental protocol approved by the University of Chicago Institutional Review Board and in accordance with the guidelines set by the Declaration of Helsinki.

### Actor-Model task

#### Stimuli and stimulus equating

After piloting (see Supplementary Material S1), we curated a sample of 90 faces for the fMRI task: 30 actors and 15 models of each gender. These stimuli were equated by independent samples of online raters (see Supplementary Material S1). For each rating dimension, we ran a 2 (Person-knowledge: well-known actors, unknown models) × 2 (Gender: male, female) repeated-measures ANOVA. The final stimuli were of average attractiveness and young to middle-aged. Males were significantly more dominant than females, but dominance did not vary by occupation. Actors were significantly more familiar and likeable than models, but neither rating varied by gender. Body-of-work ratings indicated that the work of both genders was similarly rated as average. Because the stimuli necessitated the use of photos of actors and models from unstandardized online sources, it was difficult to obtain and equate only photographs of neutral faces. Therefore, only stimuli with a happy or neutral expression were selected. However, facial expression did not statistically differ from an expected distribution of 2/3 neutral and 1/3 happy faces in each occupation-by-gender condition.

#### fMRI task design

The actor-model task consisted of an event-related design with two counterbalanced functional runs. The equated selection of 60 actors and 30 models was divided into two sets of 30 unique actors and two sets of 15 unique models, respectively. Each participant rated each of the 60 actors once on a single dimension (attractiveness or body of work) and each of the 30 models once on attractiveness.

In addition to counterbalancing the assignment of face set to runs (run 1 vs. run 2), the assignment of individual faces to evaluative rating conditions (attractiveness vs. body of work), and the assignment of response keys (ascending vs. descending), we also counterbalanced the order in which participants completed the three conditions within each run. The block orders of the two runs were completely orthogonal (i.e., no blocks were presented in the same location of the block sequence across runs). Accounting for the above factors, each participant completed one of 48 versions of the experiment. (See Supplementary Material S1 more details.)

**Figure 3:**
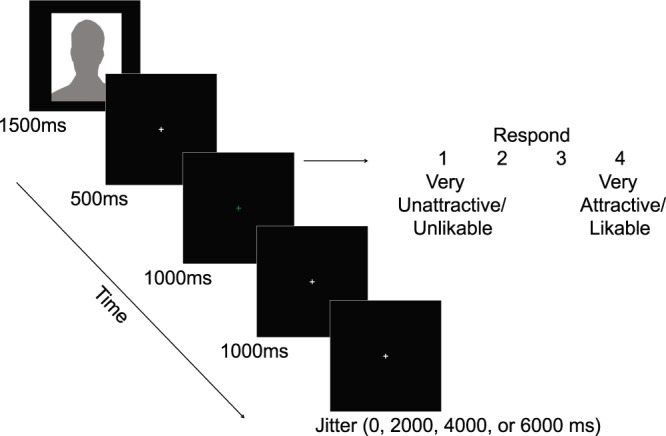
Trial sequence for the actor–model fMRI task. Over the course of the experiment (i.e., two fMRI runs), participants evaluated 30 unique actors on attractiveness, 30 unique actors on body of work, and all 30 models on attractiveness. The silhouette presented here represents a picture of either an actor or model stimulus. Each run was split into three counterbalanced blocks, each of which began with a prompt indicating the evaluative dimension and the target type (e.g., “How attractive are these actors?”).

Before each block, participants saw a brief cue consisting of the rating dimension and target occupation (e.g., “How attractive are these actors?”). All trials were presented on a black background and consisted of a face presented for 1500 ms followed by a 500-ms white fixation. Next, a green fixation appeared prompting participants to provide their rating (i.e., attractiveness or likability based on body of work: see Fig. 3). Responses were given on a counterbalanced four-point scale. After 1000 ms, the green fixation was replaced by a final 1000-ms white fixation. Altogether, each trial lasted 4000 ms, and participants had a window from 1500–3500 ms post-stimulus onset to both form their evaluation and respond using the button box. Post-trial jittering was pseudorandomly interleaved, using 0-, 2000-, 4000-, or 6000-ms fixations (Fig. 3). Stimulus presentation and data collection were in E-Prime 2.0 Professional (www.pstnet.com/eprime).

### Procedure

In the context of a larger study, participants completed online demographic and unrelated individual differences questionnaires at home (see Supplementary Material S3). (For all experiments, we have reported all measures, conditions, data exclusions, and sample size determinations either in the main text or supplementary material.) Then participants received a list of the actors with links to their online profiles along with instructions to study the actors. Prior to coming into the lab for scanning, participants had to get 100% correct on an online quiz in which they had to identify one of two movies or write in a different movie casting that actor. This was done to ensure the participants were familiar with the actors’ body of work.

Upon arriving for their scanning session, participants gave informed consent and received task instructions. Outside of the scanner, participants then completed a practice block consisting of a shortened version of the main experiment (one run consisting of one 10-trial block for each of the three conditions) conducted on a computer with actors and models that would not appear in the actual study. Participants were then trained on an impression-formation task unrelated to the current investigation^22,55^ (see Supplementary Material S1 for full procedures).

Participants then completed the actor-model task in the scanner. After this, participants completed two runs of the unrelated impression-formation task. Finally, resting-state and anatomical scans were acquired if time allowed. Post-scan, participants completed questionnaires unrelated to the present investigation (see Supplementary Material S3). In order to verify that our participants were indeed familiar with the actors and unfamiliar with the models (who were selected to be unfamiliar based on ratings from an independent sample), participants indicated their familiarity with all actors and models viewed in the scanner on a 9-point scale from 1 “Extremely Unfamiliar” to 9 “Extremely Familiar”. For analyses that parallel those in the results section but while accounting for these familiarity ratings, see Supplementary Material S2.

### Data analysis

#### Trial- and participant-level exclusions

For each participant with complete fMRI and behavioral data sets (*n* = 58), we excluded from analyses: (1) all trials in which the participant did not provide an in-scanner rating, (2) all actors that the participant rated as somewhat to very unfamiliar (i.e., 1–5 on the post-scan familiarity ratings), and (3) all models that the participant rated as appearing somewhat to very familiar (i.e., 4–9 on the post-scan familiarity ratings) (see Supplementary Material S1 for exclusions). Only 58 out of 104,400 trials were eliminated due to missing in-scanner ratings. After all trial-level exclusions, three participants were ultimately eliminated for having fewer than six trials per rating dimension (i.e., actors rated on attractiveness, models rated on attractiveness, and actors rated on body of work). The remaining 55 participants’ data were analyzed. The means and standard deviations of the three in-scanner rating conditions were as follows: actors rated on attractiveness, *M* = 2.907, *SD* = 0.414; models rated on attractiveness, *M* = 2.762, *SD* = 0.339; and actors rated on body of work, *M* = 3.142, *SD* = 0.318. These mean ratings were all significantly different from each other (see Supplementary Material S1 for full statistics). Within participants, we generally observed similar variability (i.e., participant-specific standard deviations in trial-level responses) for each of the three conditions, *F*(2,159) = 0.376, *p* = 0.687: actors rated on attractiveness, *M*_*SD*_ = 0.825; models rated on attractiveness, *M*_*SD*_ = 0.837; and actors rated on body of work, *M*_*SD*_ = 0.856.

#### fMRI data acquisition and analysis

Anatomical and functional whole-brain imaging were performed using a Philips Achieva 3.0-T scanner and 32-channel head coil at the University of Chicago MRI Research Center. Functional images in the form of 30 oblique slices were collected using Z-shim acquisition in 2 runs of 163 TRs each, using pulse sequence parameters (TR/TE = 2000/25 ms, flip angle = 77°, interleaved slices with 4.0-mm thickness, 0.5-mm gap, FOV = 192 × 134 × 192 mm, approximately 64 × 64-mm matrix). The order of slice acquisition varied across participants, depending on the location of the four z-shim slices^56^. High-resolution T1-weighted anatomical images were acquired in the sagittal plane using a 3D Turbo Field Echo (TFE/MP-RAGE) anatomical scan (TR = 8.0 ms, TE = 3.5 ms, FOV = 228 × 240 × 181 mm, 1.0-mm slice thickness, no gap, 240 × 240-mm matrix). Then thin-slice resting-state scans were collected (TR = 2000 ms, TE = 29.5 ms, FOV = 240 × 138 × 240 mm, slice thickness = 2.6 mm with a 1.4-mm gap, an in-plane resolution of 3.75 mm^2^, and a flip angle of 77°).

***GLM***. Functional MRI data were analyzed using general linear models (GLM) in SPM8 (Wellcome Department of Cognitive Neurology, London, UK). Analyses were facilitated by a custom suite of scripts for fMRI analysis (https://github.com/ddwagner/SPM8w). Data were preprocessed to remove sources of noise and artifacts. Images were realigned within and across runs to correct for head movement. Functional data were transformed into a standard anatomical space (3-mm isotropic voxels) based on the ICBM 152 brain template (Montreal Neurological Institute). Normalized data were then spatially smoothed using an 8-mm Gaussian kernel to increase signal-to-noise ratio and to reduce the impact of anatomical variability that was not corrected by stereotaxic normalization.

For each participant, a GLM was constructed to examine condition-specific brain activity as a function of the participant’s in-scanner ratings (either attractiveness or likability of body of work). In-scanner ratings were coded such that larger numbers represented more positive evaluations. We used these ratings to model three regressors, each representing a linear change in neural responses within a given rating condition as condition-specific ratings increased. These regressors were incorporated as parametric predictors in each participant’s level-1 GLM. Our reported analyses focused on these three parametric predictors of interest: (1) actors evaluated on body of work (i.e., familiar others rated on person-knowledge), (2) actors evaluated on attractiveness (i.e., familiar others rated on perceptual cues), and (3) models evaluated on attractiveness (i.e., unfamiliar others rated on perceptual cues). In sum, one single GLM incorporating three regressors for each of the three conditions (i.e., non-parametric parameters), three regressors for the condition-specific parametric parameters, and additional regressors for covariates of non-interest (a session mean, a linear trend to account for low-frequency drift, and six movement parameters derived from realignment corrections) were convolved with a canonical hemodynamic response function and used to compute parameter estimates (*β*) for each condition at each voxel. Resulting estimates for each participant were used in ROI analyses and exploratory whole-brain parametric analyses.

#### VMPFC ROI

ROI analyses were conducted to identify the hypothesized impact of person-knowledge and perceptual cues on evaluations. Analyses focused on an 8-mm VMPFC spherical ROI that was centered on the MNI coordinates from a previous research examining various forms of person-knowledge (e.g., financial and moral status^23,41^, socio-economic status^22^, and moral appraisals^57^) during social evaluations (0, 52, −6). For each participant, mean GLM parameter estimates for the VMPFC ROI were extracted for each of the three parametric predictors of interest. ROI analyses were conducted offline in R using the stats package^58^. We first conducted one-sample t-tests to compare each parametric predictor to zero to explore whether VMPFC activity changed as a function of ratings (i.e., increased as evaluations became more positive) for each of the three conditions. Using the R function lm, a second analysis focused on the relative impact of each parametric predictor on VMPFC activity. Following up on a significant one-way ANOVA, we tested all possible pair-wise contrasts between parametric predictors by conducting three separate linear regressions each using a unique set of contrast codes: (1) parametric predictor for actors’ body of work = 0.5, parametric predictor for actors’ attractiveness = 0, and parametric predictor for models’ attractiveness = −0.5; (2) parametric predictor for actors’ body of work = 0, parametric predictor for actors’ attractiveness = 0.5, and parametric predictor for models’ attractiveness = −0.5; and (3) parametric predictor for actors’ body of work = 0.5, parametric predictor for actors’ attractiveness = −0.5, and parametric predictor for models’ attractiveness = 0).

### Exploratory and supplementary analyses

In addition to our main focus on the VMPFC, we conducted exploratory analyses on other regions of interest previously suggested to be involved in person evaluation and social cognition: DMPFC, bilateral nucleus accumbens, bilateral superior temporal sulcus, bilateral temporo-parietal junction, precuneus, and bilateral amygdala (see Supplementary Material S2).

Additionally, we conducted exploratory whole-brain analyses (see Results) to identify additional brain regions potentially sensitive to modulation by in-scanner ratings in each of the three conditions: (1) person-knowledge use (i.e., actors rated on body of work only), (2) person-knowledge availability (i.e., actors rated on attractiveness only), and (3) percept-based evaluations without person-knowledge (i.e., models rated on attractiveness only).

Finally, we also conducted supplementary ROI and whole-brain analyses controlling for post-scan ratings of stimulus familiarity in order to control for any remaining effects of familiarity (see Supplementary Material S2).

## Supplementary information

Supplementary Material

## Data Availability

Datasets generated and/or analyzed during the current study are available from the corresponding author upon request.
