# Supplementary material for: The ventromedial prefrontal cortex is particularly responsive to social evaluations requiring the use of person-knowledge
Source: Sci Rep. 2019 Mar 25;9:5054. doi: 10.1038/s41598-019-41544-z (PMC6434022; doi:10.1038/s41598-019-41544-z)
Supplement: Supplementary file 1 — Supplementary Material [file 41598_2019_41544_MOESM1_ESM.pdf]

The ventromedial prefrontal cortex is particularly responsive to social evaluations requiring the use of person-knowledge

Tzipporah P. Dang[1†], Bradley D. Mattan[1†], Jennifer T. Kubota[1, 2], and Jasmin Cloutier[1*]

† Shared First Authorship

Affiliations:

[1] Department of Psychological and Brain Sciences, University of Delaware, Newark, USA

[2] Department of Political Science and International Relations, University of Delaware, Newark, USA

Corresponding author:

Jasmin Cloutier

Department of Psychological and Brain Sciences

University of Delaware

108 Wolf Hall

Newark, DE 19716

Telephone: +1 (302) 831-4813

Fax: +1 (302) 831-6393

Email: jcloutier@psych.udel.edu

S1: Supplementary Method

We provide a supplementary overview of aspects of the protocol that were pertinent to the current study and the unrelated fMRI study on perceived race and social status[1,2].

Screening Criteria and Procedures

Because this study was run together with a study investigating perceived race, only White participants were invited to participate in this experiment. Beyond this race-related requirement, participants were eligible to participate if they met the following inclusion criteria: (1) between 18 and 35 years old, (2) have lived in the U.S. for at least 5 years, (3) have a good command of the English language, (4) no history of drug abuse, (5) right handed, (6) no history of serious head injury, (7) no color vision problems, (8) no current acute illness, (9) not currently taking psychotropic medication, (10) no diagnosis of developmental disorders, (11) no diagnosis of a chronic disease that compromises mental, neural, or autonomic function, and (12) passed an MRI safety screen. Of the 82 eligible screened participants, 61 completed the study from start to finish. The remaining 21 screened participants failed to complete the required online pre-test measures and/or later opted to discontinue their participation.

Stimulus Piloting

Three hundred pictures of White male and female actors and models were collected from actor profiles or online modeling agencies: 100 male actors, 100 female actors, 50 male models, and 50 female models. The targets displayed directed eye gaze, were cropped around the hair and from the neck up using Adobe Photoshop, and placed on grey backgrounds using the SHINE toolbox [3] The stimuli were vertically and horizontally centered on the grey background, occupying 100% of the background height and 75% of the background width. Separate groups of participants on Amazon Mechanical Turk (Mturk) rated stimuli on various dimensions: attractiveness (*n*=28), likability (*n*=22), dominance (*n*=30), familiarity (*n*=18), facial expression

and expression intensity (n=31), age (n=32), body of work quality (n=30), and occupation identification (n=31). Ratings for attractiveness were provided on a 7-point scale with 1 as "not attractive at all" to 7 as "very attractive." Ratings for likability were provided on a 7-point scale with 1 as "not likable at all" to 7 as "very likable." Ratings for dominance were provided on a 7-point scale with 1 as "not dominant at all" to 7 as "very dominant." Ratings for familiarity were provided on a 7-point scale with 1 as "not at all familiar" to 7 as "very familiar." Ratings for facial expression were categorical (i.e., angry, happy, neutral, and sad) and ratings for facial expression intensity were provided on a 5-point scale from "not intense at all" to "extremely intense." Ratings for age in years were categorical (i.e., 18–25, 26–35, 36–45, 46–55, 56–65) with numbered responses from 1–5 associated with each age range. Additionally, for only actors, ratings for body of work (i.e., the actor's or actress's accomplishments in cinema and television programming) were provided on a 7-point scale with 1 as "not at all impressive" to 7 as "very impressive." Occupation identification was assessed with a binary measure (i.e., "Yes, this person is an actor" or "No, this person is not an actor"). We only selected actors for further equating if they were correctly identified as actors by at least 60% of online raters.

Each rating was analyzed using a 2 (Person-knowledge: well-known actors, unknown models) × 2 (Gender: male, female) repeated-measures ANOVA. Findings for each rating are summarized in the following paragraphs.

Final stimuli in each condition were of average attractiveness, M=4.530, all $F(1,27)<0.050$, all $p>.825$, and on average young to middle-aged, M=2.594, all $F(1,31)<1.075$, all $p>.307$.

Males were rated significantly more dominant than females, M_{Male}=4.471, M_{Female}=4.167, $F(1,29)=7.832$, $p=.009$. However, dominance did not vary by occupation, and there was no Person-knowledge × Gender interaction, all $F(1,29)<0.703$, all $p>.408$.

As anticipated, actors were rated as significantly more familiar than models: M_{Actor}=4.255, M_{Model}=2.085, $F(1,17)$=49.870, p<.001. This effect was qualified by a Person-knowledge × Gender interaction, $F(1,17)$=4.444, p=.050. Follow-up contrasts indicated that actors were more familiar than models for both females, $M_{FemaleActor}$=4.128, $M_{FemaleModel}$=2.182, $t(17)$=6.003, p<.001, and males, $M_{MaleActor}$=4.382, $M_{MaleModel}$=1.989, $t(17)$=7.347, p<.001, with the magnitude of the person-knowledge effect being numerically larger for males than for females. Familiarity within each occupation did not vary reliably by gender, $|t(17)|$<1.764, p>.095.

Actors were rated significantly more likeable than models, M_{Actors}=4.482, M_{Models}=4.268, $F(1,21)$=6.983, p=.015. However, likability did not vary by gender, and there was no Person-knowledge × Gender interaction, all $F(1,21)$<0.487, all p>.492.

Body-of-work ratings for actors indicated average impressiveness for the work of both female and male actors, M=4.162, $t(29)$=-1.136, p=.265.

For facial expression, we selected stimuli that at least 70% of raters categorized as having a happy or neutral expression. Facial expression did not statistically differ from the expected hypothesis of 2/3 neutral and 1/3 happy faces in each occupation-by-gender condition, χ^2 (7, n=90)=5.400, p=.611.

We also calculated a facial expression intensity score for the male and female actors and models. Facial intensity scores varied by person-knowledge, $F(1,30)$=52.067, p<.001, and gender, $F(1,30)$=15.378, p<.001. These effects were qualified by a Person-knowledge × Gender interaction, $F(1,30)$=11.369, p=.002, in which facial expression intensity was greater for female actors (M=11.073) compared to male actors (M=9.229), $t(30)$=5.300, p<.001; for female actors (M=11.073) compared to female models (M=11.905), $t(30)$=-2.392, p=.023; and for male actors (M=9.229) compared to male models (M=11.675), $t(30)$=-7.867, p<.001.

Pre-Scan Procedure

Cover story and pre-scan survey. After consent forms and imaging center paperwork, the experimenter took each participant's picture using a digital camera and tripod. It was explained to the participant that he would later take part in a trust game unrelated to the current study for a chance to earn more money. The trust game included responses from real participants paired with their photographs. The experimenter further explained that the participant's responses in the trust game would be paired with his own picture and used in a future study with other participants. Participants then completed the State and Trait Anxiety, the Beck Depression Inventory II, and stress/anxiety sliding scales (see Supplementary Material S3).

Race–status task training. The race–status task training was based on training procedures used in previous work[1,4].

Actor-Model Task Design and Exclusions

Design. As mentioned in the main text, the actor-model task consisted of an event-related design with two functional runs. The equated selection of 60 actors and 30 models was divided into two sets of 30 unique actors and two sets of 15 unique models, respectively. These unique sets of faces were divided across two functional acquisition runs, with 30 unique actors and 15 unique models in each run. Run assignment (first vs. second) for each unique set of faces was counterbalanced across participants. In each run, participants rated half of the actors on attractiveness and the other half of the actors on body of work. The halves of each actor set to be rated on attractiveness versus body of work were counterbalanced across participants. In summary, each participant rated each of the 60 actors once on a single dimension and each of the 30 models once on attractiveness. Finally, key assignment for responding was counterbalanced such that half of participants responded on a scale of 1 (very attractive/likable) to 4 (very unattractive/unlikable) and in reverse assignment for the other half.

In addition to counterbalancing the assignment of face set to runs (run 1 vs. run 2), the assignment of individual faces to evaluative rating conditions (attractiveness vs. body of work), and the assignment of response keys (ascending vs. descending), we also counterbalanced the order in which participants completed the three evaluative rating blocks within each run. The first run always used a different block ordering (e.g., actors rated on attractiveness, actors rated on body of work, and models rated on attractiveness) than the second run (e.g., models rated on attractiveness, actors rated on attractiveness, and actors rated on body of work). Specifically, the block orders of the two runs were completely orthogonal (i.e., no blocks were presented in the same location of the block sequence across runs). Following these constraints, we generated six orthogonal pairs of block orders. Taking into account all of the above factors (i.e., assignment of face set over two runs, the assignment of faces to two evaluative rating conditions, two response key assignments, and six block orderings) resulted in 48 versions of the experiment. Each participant completed one version of the experiment, with as few repeated versions as possible in the final participant sample.

Trial- and participant-level exclusions. A total of 56 trials were eliminated because no ratings were provided. We then performed the familiarity exclusions (see main text) to ensure that each participant had sufficient knowledge of the actors from which to make body-of-work evaluations and no existing knowledge about the models, which were intended to be unfamiliar. Altogether, this resulted in the removal of 390 familiar model trials (M=5.828, SD=6.532) and 1450 non-familiar actor trials: 720 in the attractiveness rating condition (M=12.276, SD=6.469) and 730 in the body-of-work rating condition (M=12.310, SD=6.545).

After these trial-level exclusions, three participants were ultimately eliminated for having fewer than six trials per rating dimension (i.e., actors rated on attractiveness, models rated on attractiveness, and actors rated on body of work). The mean post-scan familiarity scores of the

models (*M*=1.498) and actors (*M*=7.864) included in our analyses were significantly different from each other, $t(77)=112.150$, $p<.001$.

Mean and variance for ratings after exclusions. In order to test for differences in means of in-scanner ratings across conditions, we computed a repeated-measures ANOVA on the means for each of the three conditions: Mean ~ Condition + Error(Subject/(Condition)). The mean ratings by condition were significantly different from one other, $F(2,159)=5.029$, $p=.008$. Paired t-tests revealed that parameter estimates for all three conditions differed significantly from one another. Actors were rated more positively on their body of work (*M*=3.142, *SD*=0.318) than on their attractiveness (*M*=2.907, *SD*=0.414), $t(54)=4.312$, $p<.001$. Actors (*M*=2.907, *SD*=0.414) were rated as more attractive than models (*M*=2.762, *SD*=0.339), $t(54)=3.116$, $p=.003$. Actors rated on body of work (*M*=3.142, *SD*=0.318) were rated more positively than models rated on attractiveness (*M*=2.762, *SD*=0.339), $t(54)=6.872$, $p<.001$.

In order to test for differences in variance of in-scanner ratings within-participant by condition, we computed a repeated measures ANOVA on the standard deviation scores by subject for each of the three conditions: SD ~ Condition + Error(Subject/(Condition)). The mean standard deviations for all three conditions were not significantly different from one another, $F(2,159)=0.376$, $p=.687$: actors rated on body of work ($M_{SD}=0.856$), actors rated on attractiveness ($M_{SD}=0.825$), and models rated on attractiveness ($M_{SD}=0.837$).

Race–Status fMRI Experiment

Following the actor–model fMRI scan, participants completed a race–status impression formation task in the scanner[1,2].

Post-Scan Procedure

Following their scan, participants completed explicit rating measures including a social distance task, likeability ratings, status recall, the trust game, and the actor–model familiarity

task. Finally, participants completed questionnaires including ratings of their present basic emotions, the PANAS, the Perceived Stress Scale, a measure of chronic stress, and a measure of social value orientation. See Supplementary Material S3 for further details on these measures. Then after their final saliva sample, participants were paid and debriefed.

Saliva Sample Acquisition

Saliva samples were collected for the race–status experiment[1]. Upon arrival, participants received water to facilitate saliva collection. Ten minutes after, the experimenter collected the first baseline sample. An additional baseline sample was acquired immediately prior to scanning. Two post-scan samples were acquired approximately 66 and 109 minutes after the second baseline sample.

S2: Supplementary Analyses

Exploratory ROI Analyses

Analysis parameters. As mentioned in the main text, we also conducted an exploratory examination of ROIs implicated in the use of person-knowledge and mentalizing[5] [dorsal medial prefrontal cortex (DMPFC); MNI: -3, 55, 23 [6]], reward processing of attractive faces[7,8] [bilateral nucleus accumbens, NAcc; left NAcc, MNI: -9, 11, -13 [9]; right NAcc, MNI: 10, 18,-10 [9]], mentalizing[10,11] [bilateral superior temporal sulcus (STS); left STS, MNI: -59, -15, -16 [6]; right STS, MNI: 57, -10, -20 [6] and bilateral temporo-parietal junction (TPJ); left TPJ, MNI: -56, -55, 16 [6]; right TPJ, MNI: 54, -51, 17 [6]], perceptual familiarity[5] [precuneus; MNI: -5, -55, 32 [6]], and processing of evaluative relevant stimuli[12] [bilateral amygdala; left amygdala, MNI: -24, -6, -24 [13]; right amygdala, MNI: 18, -6, -21 [13]]. These analyses were the same as those reported in the main text for the VMPFC.

We first conducted one-sample t-tests to compare each parametric predictor to zero to explore whether activity in each exploratory ROI changed as a function of ratings (i.e., increased as evaluations became more positive) for each of the three conditions. Using the R function lm, a second analysis focused on the relative impact of each parametric predictor on activity in each exploratory ROI. Specifically, to compare the differences between parametric predictors, we conducted three separate linear regressions each using a unique set of contrast codes: (1) parametric predictor for actors' body of work = .5, parametric predictor for actors' attractiveness = 0, and parametric predictor for models' attractiveness = -.5; (2) parametric predictor for actors' body of work = 0, parametric predictor for actors' attractiveness = .5, and parametric predictor for models' attractiveness = -.5; and (3) parametric predictor for actors' body of work = .5, parametric predictor for actors' attractiveness = -.5, and parametric predictor for models' attractiveness = 0). However, for some of the exploratory ROIs reported here (i.e., bilateral

NAcc, right STS, and bilateral TPJ), the contrast codes for the linear regressions differed from what was used for the VMPFC because the pattern of parametric modulation differed from the observed linear pattern in the VMPFC. In other words, parametric modulation for actors rated on body of work was not always larger than parametric modulation for actors rated on attractiveness, which was not always larger than for models rated on attractiveness. Any contrast coding that is different from the coding used for the VMPFC in the main text is explicitly stated in this section.

Do evaluations based on person-knowledge or attractiveness modulate exploratory ROI activity? We first examined whether increased positive evaluations led to greater exploratory ROI activity when those evaluations were based on (1) perceptual cues without person-knowledge (i.e., models rated on attractiveness), (2) perceptual cues with available person-knowledge (i.e., actors rated on attractiveness), and (3) person-knowledge (i.e., actors rated on body of work). Separate analyses were conducted for DMPFC, bilateral NAcc, bilateral STS, bilateral TPJ, precuneus, and bilateral amygdala. We present these results to spur future research and for meta-analytic purposes; therefore, below we report uncorrected p-values. However, readers should note that when aggregating across laterality, the Bonferroni-corrected p-value to achieve significance for these exploratory analyses would be $p<.008$.

DMPFC. All effects were non-significant, $|t(54)|<1.113$, $p>.270$. The use or availability of person-knowledge did not reliably affect DMPFC activity.

NAcc. All effects in the right NAcc were non-significant, $|t(54)|<0.879$, $p>.382$. We observed significant left NAcc involvement for actors rated on likability based on body of work, $t(54)=2.515$, $p=.015$, such that left NAcc activity increased as ratings of likability based on body of work became more positive. However, we did not observe significant left NAcc involvement for actors rated on attractiveness or models rated on attractiveness, $|t(54)|<0.519$, $p>.605$. Taken

together, these results indicate that only the left NAcc may be sensitive to positive evaluations during the use of available person-knowledge.

STS. All effects in the left STS were non-significant, $|t(54)|<1.588$, $p>.117$. We observed significant right STS involvement for actors rated on attractiveness, $t(54)=2.261$, $p=.028$[i], such that right STS activity increased as ratings of likability based on body of work became more positive. However, we did not observe significant right STS involvement for actors rated on likability based on body of work or for models rated on attractiveness, $|t(54)|<1.943$, $p>.056$. Taken together, the left STS did not show preferential activity regardless of rating dimension while the right STS was sensitive to positive evaluations when person-knowledge was available but not when it was relevant to evaluations.

TPJ. All effects in bilateral TPJ were non-significant, $|t(54)|<1.733$, $p>.087$. The use or availability of person-knowledge did not reliably affect bilateral TPJ activity.

Precuneus. We observed significant precuneus involvement for actors rated on likability based on body of work, $t(54)=2.304$, $p=.025$[i], such that precuneus activity increased as ratings of likability based on body of work became more positive. We also observed significant precuneus involvement for actors rated on attractiveness, $t(54)=2.385$, $p=.021$[i], such that precuneus activity increased as ratings of attractiveness for actors became more positive. However, we did not observe significant precuneus involvement for models rated on attractiveness, $t(54)=0.958$, $p=.342$. Taken together, these results indicate that the precuneus may be sensitive to positive evaluations when person-knowledge is available, irrespective of whether that person-knowledge is directly relevant to evaluations.

---

[i] Note that this contrast does not survive Bonferroni correction for multiple comparisons: $\alpha = .0167$.

Amygdala. We observed significant left amygdala involvement for actors rated on likability based on body of work, $t(54)=2.677$, $p=.010$, such that left amygdala activity increased as ratings of likability based on body of work became more positive. We also observed significant left amygdala involvement for actors rated on attractiveness, $t(54)=2.103$, $p=.040$[i], such that left amygdala activity increased as ratings of attractiveness for actors became more positive. However, we did not observe a significant left amygdala involvement for models rated on attractiveness, $t(54)=-0.225$, $p=.823$. Additionally, we observed significant right amygdala involvement for actors rated on attractiveness, $t(54)=2.149$, $p=.036$[i], such that right amygdala activity increased as ratings of likability based on body of work became more positive. However, we did not observe significant right amygdala involvement for actors rated on likability based on body of work or for models rated on attractiveness, $|t(54)|<1.076$, $p>.286$. Taken together, these results indicate that whereas the left amygdala is sensitive to positive evaluations in the presence of person-knowledge regardless of its relevance to evaluations, this is the case for the right amygdala only when that person-knowledge is relevant to evaluations.

Do person evaluations modulate exploratory ROI activity more when person-knowledge is used or when it's simply available? To examine whether sensitivity to positive ratings was especially pronounced for a particular condition (e.g., use of person-knowledge: actors' body of work) relative to other conditions (e.g., mere presence of person-knowledge: actors' attractiveness), we next conducted a linear regression on parameter estimates to test for differences between all parametric predictors. As above, separate analyses were conducted for DMPFC, bilateral NAcc, bilateral STS, bilateral TPJ, precuneus, and bilateral amygdala.

DMPFC. The results did not reveal significant differences among parametric predictors for DMPFC activity, $F(2,162)=0.661$, $p=.518$. Additionally, no significant differences emerged

when considering linear changes in DMPFC activity as a function of parametric predictors (all $p>.259$).

NAcc. The results did not reveal a significant differences among parametric predictors for left NAcc activity, $F(2,162)=2.482$, $p=.087$. However, because we were particularly interested in the impact of the use and availability of person-knowledge, we nonetheless conducted a linear regression on parameter estimates comparing linear changes in parametric predictors. Results revealed a significant linear relationship between person-knowledge use and availability (contrast codes: parametric predictor for actors' body of work = .5, parametric predictor for actors' attractiveness = -.5, and parametric predictor for models' attractiveness = 0), such that left NAcc activity was greater as ratings of likability based on body of work for actors became more positive compared to when ratings of attractiveness for actors became more positive, $b=.209$, $SE=.097$, $CI_{95\%}=[.019, .398]$, $F(1,163)=4.667$, $p=.032$[i]. No other significant differences between parametric predictor magnitudes emerged: (1) ratings of likability based on body of work for actors compared to ratings of attractiveness for models, $b=.152$, $SE=.097$, $CI_{95\%}=[-.039, .342]$, $F(1,163)=2.482$, $p=.121$; and (2) ratings of attractiveness for models compared to ratings of attractiveness for actors, $b=.057$, $SE=.098$, $CI_{95\%}=[-.135, .249]$, $F(1,163)=0.341$, $p=.560$.

Additionally, the results did not reveal any significant differences among parametric predictors for right NAcc activity, $F(2,162)=0.023$, $p=.977$. No significant differences emerged when considering linear changes in NAcc activity as a function of parametric condition (all $p>.848$). Taken together, these results indicate that left NAcc may be sensitive to positive evaluations based on person-knowledge (vs. perceptual attributes).

STS. The results did not reveal significant differences among parametric predictors for left STS activity, $F(2,162)=0.972$, $p=.380$ or right STS activity $F(2,162)=1.032$, $p=.359$.

Additionally, no significant differences emerged when considering linear changes in either left or right STS activity as a function of parametric predictors (all p>.176).

TPJ. The results did not reveal significant differences among parametric predictors for left, $F(2,162)=0.414$, $p=.662$, or right, $F(2,162)=0.199$, $p=.820$, TPJ activity. Additionally, no significant differences emerged when considering linear changes in either left or right TPJ activity as a function of parametric predictors (all p>.292).

Precuneus. The results did not reveal significant differences among parametric predictors for precuneus activity, $F(2,162)=1.166$, $p=.314$. Additionally, no significant differences emerged when considering linear changes in precuneus activity as a function of parametric predictors (all p>.139).

Amygdala. The results did not reveal a significant differences among parametric predictors for left amygdala activity, $F(2,162)=2.578$, $p=.082$. However, because we were particularly interested in the impact of the use and availability of person-knowledge, we nonetheless conducted a linear regression on parameter estimates comparing all parametric predictors. As in the VMPFC (see main text) and right amygdala (see below), the results revealed a significant linear relationship between positive ratings and the combined availability/use of person-knowledge, such that left amygdala activity was greater as ratings of likability based on body of work for actors became more positive compared to when ratings of attractiveness for models became more positive, $b=.363$, $SE=.167$, $CI_{95\%}=[.037, .690]$, $F(1,163)=4.763$, $p=.031$[i]. No other significant differences emerged: (1) ratings of likability based on body of work for actors compared to ratings of attractiveness for actors, $b=.099$, $SE=.169$, $CI_{95\%}=[-.232, .429]$, $F(1,163)=0.341$, $p=.560$; and (2) ratings of attractiveness for actors compared to ratings of attractiveness for models , $b=.265$, $SE=.168$, $CI_{95\%}=[-.064, .593]$, $F(1,163)=2.500$, $p=.116$.

For right amygdala activity, the results revealed significant differences among parametric predictors, $F(2,162)=3.057$, $p=.050$[ii]. As in the VMPFC (see main text) and left amygdala (see above), the results revealed a significant linear relationship between positive ratings and the combined availability/use of person-knowledge (contrast codes: parametric actor body of work = .5, parametric actor attractiveness = 0, and parametric model attractiveness = -.5), such that right amygdala activity was greater as ratings of likability based on body of work for actors became more positive compared to when ratings of attractiveness for models became more positive, $b=.433$, $SE=.175$, $CI_{95\%}=[.091, .776]$, $F(1,163)=6.150$, $p=.014$. No other significant differences emerged: (1) ratings of likability based on body of work for actors compared to ratings of attractiveness for actors, $b=.211$, $SE=.178$, $CI_{95\%}=[-.136, .558]$, $F(1,163)=1.417$, $p=.236$; and (2) ratings of attractiveness for actors compared to ratings of attractiveness for models , $b=.222$, $SE=.177$, $CI_{95\%}=[-.125, .570]$, $F(1,163)=1.576$, $p=.211$. Taken together, these results indicate that, like the VMPFC (see main text), bilateral amygdala may be sensitive to the use of available person-knowledge relative to the absence of person-knowledge.

Supplementary Analyses Controlling for Post-Scan Familiarity Ratings

As mentioned in the main text, supplementary analyses were conducted in order to determine whether the VMPFC and the exploratory ROIs were robust to post-scan familiarity ratings. In these supplementary analyses, we therefore controlled for post-scan familiarity ratings to ensure that any remaining variability in familiarity could not account for the effects. Specifically, we re-analyzed the ROI data while accounting for individual ratings of familiarity as a parametric modulator in the level-1 GLM. In sum, one single GLM incorporating three regressors for each of the three conditions (i.e., non-parametric parameters), three regressors for

[ii] This value was rounded up from $p=.04975$.

the condition-specific parametric parameters, three regressors for individual ratings of familiarity, and additional regressors for covariates of non-interest (a session mean, a linear trend to account for low-frequency drift, and six movement parameters derived from realignment corrections) were convolved with a canonical hemodynamic response function and used to compute parameter estimates (β) for each condition at each voxel. Results from these analyses were consistent with those reported when not accounting for post-scan ratings in familiarity, except for bilateral amygdala.

Finally, in order to account for individual ratings of familiarity in the whole-brain exploratory analyses, we also re-analyzed these data including post-scan familiarity ratings as a parametric modulator in the level-1 GLM for each whole-brain analysis. Results from the whole brain analyses were also generally consistent.

Do evaluations based on person-knowledge or attractiveness modulate activity in the VMPFC and exploratory ROIs? Again, we first examined whether increased positive evaluations led to greater exploratory ROI activity when those evaluations were based on (1) perceptual cues without person-knowledge (i.e., models rated on attractiveness), (2) perceptual cues with available person-knowledge (i.e., actors rated on attractiveness), and (3) person-knowledge (i.e., actors rated on body of work). As in the previous section, we did this by conducting separate one-sample t-tests on parameter estimates for each parametric predictor (i.e., actors rated on likability based on body of work, actors rated on attractiveness, and models rated on attractiveness) compared to zero.

VMPFC. Controlling for post-scan familiarity ratings, we found similar findings in VMPFC as reported in the main text. We observed a significant effect in the VMPFC for actors rated on likability based on body of work, $t(54)=3.726$, $p<.001$, such that VMPFC activity increased as ratings of likability based on body of work became more positive. We also observed

significant VMPFC involvement for actors rated on attractiveness, $t(54)=2.585$, $p=.012$, such that VMPFC activity increased as ratings of attractiveness for actors became more positive. However, we did not observe significant VMPFC involvement for models rated on attractiveness, $t(54)=0.506$, $p=.615$. Taken together, these results indicate that the VMPFC is sensitive to the availability of person-knowledge, irrespective of rating dimension.

DMPFC. When controlling for post-scan familiarity, we observed similar findings in DMPFC as when we did not control for post-scan familiarity ratings. All effects were non-significant, $|t(54)|<1.029$, $p>.306$. The use or availability of person-knowledge did not reliably affect DMPFC activity.

NAcc. When controlling for post-scan familiarity, we observed similar findings in NAcc as when we did not control for post-scan familiarity ratings. Specifically, we observed significant left NAcc involvement for actors rated on likability based on body of work, $t(54)=2.418$, $p=.019$[i], such that left NAcc activity increased as ratings of likability based on body of work became more positive. However, we did not observe significant left NAcc involvement for actors rated on attractiveness and models rated on attractiveness, $|t(54)|<0.785$, $p>.435$. All effects in the right NAcc were non-significant, $t(54)<1.205$, $p>.233$. Taken together, these results indicate that left NAcc may be sensitive to the use of available person-knowledge.

STS. When controlling for post-scan familiarity, we observed similar findings in STS as when we did not control for post-scan familiarity ratings. All effects in the left STS were non-significant, $|t(54)|<1.410$, $p>.163$. We observed significant right STS involvement for actors rated on likability based on body of work, $t(54)=2.032$, $p=.047$[i], such that right STS activity increased as ratings of likability based on body of work became more positive. We also observed significant right STS involvement for actors rated on attractiveness, $t(54)=2.295$, $p=.026$[i], such that right STS activity increased as ratings of attractiveness for actors became more positive.

However, we did not observe significant right STS involvement for models rated on attractiveness, $t(54)=0.643$, $p=.523$. Taken together, these results indicate that the right STS may be sensitive to the availability of person-knowledge, irrespective of its relevance to evaluations.

TPJ. When controlling for post-scan familiarity, we observed similar findings in TPJ as when we did not control for post-scan familiarity ratings. All effects in bilateral TPJ were non-significant, $|t(54)|<1.702$, $p>.094$. The use or availability of person-knowledge did not reliably affect bilateral TPJ activity.

Precuneus. When controlling for post-scan familiarity, we observed similar findings in precuneus as when we did not control for post-scan familiarity ratings. We observed significant precuneus involvement for actors rated on likability based on body of work, $t(54)=2.419$, $p=.019$[i], such that precuneus activity increased as ratings of likability based on body of work became more positive. We also observed significant precuneus involvement for actors rated on attractiveness, $t(54)=2.223$, $p=.030$[i], such that precuneus activity increased as ratings of attractiveness for actors became more positive. However, we did not observe significant precuneus involvement for models rated on attractiveness, $t(54)=0.823$, $p=.414$. Taken together, these results indicate that the precuneus may be sensitive to the availability of person-knowledge, irrespective of its relevance to evaluation.

Amygdala. When controlling for post-scan familiarity, we observed different findings in amygdala activity compared to when we did not control for post-scan familiarity ratings. All effects in bilateral amygdala were non-significant, $|t(54)|<1.784$, $p>.079$. The use or availability of person-knowledge did not reliably affect bilateral amygdala activity when accounting for individual familiarity ratings.

Do person evaluations modulate activity in the VMPFC and exploratory ROIs more when person-knowledge is used or when it's simply available? To examine whether

sensitivity to positive ratings was especially pronounced for a particular condition (e.g., use of person-knowledge: actors' body of work) relative to other conditions (e.g., mere presence of person-knowledge: actors' attractiveness), we next conducted a linear regression on parameter estimates to test for differences between all parametric predictors. As above, separate analyses were conducted for VMPFC (confirmatory) and DMPFC, bilateral NAcc, bilateral STS, bilateral TPJ, precuneus, and bilateral amygdala (exploratory).

VMPFC. When controlling for post-scan familiarity, we observed similar findings in VMPFC as when we did not control for post-scan familiarity ratings. The results revealed significant differences among parametric predictors for VMPFC activity, $F(2,162)=3.830$, $p=.024$. The results revealed a significant linear relationship between positive ratings and the combined availability/use of person-knowledge (contrast codes: parametric predictor for actors' body of work = .5, parametric predictor for actors' attractiveness = 0, and parametric predictor for models' attractiveness = -.5), such that VMPFC activity was greater as ratings of likability based on body of work for actors became more positive compared to when ratings of attractiveness for models became more positive, $b=.799$, $SE=.291$, $CI_{95\%}=[.229, 1.369]$, $F(1,163)=7.551$, $p=.007$. No other significant differences emerged: (1) ratings of likability based on body of work for actors compared to ratings of attractiveness for actors, $b=.302$, $SE=.297$, $CI_{95\%}=[-.279, .884]$, $F(1,163)=1.039$, $p=.310$; and (2) ratings of attractiveness for actors compared to ratings of attractiveness for models , $b=.497$, $SE=.295$, $CI_{95\%}=[-.081, 1.075]$, $F(1,163)=2.838$, $p=.094$. Taken together, these results indicate that the VMPFC may be sensitive to the use of available person-knowledge relative to the absence of person-knowledge.

DMPFC. When controlling for post-scan familiarity, we observed similar findings in DMPFC as when we did not control for post-scan familiarity ratings. The results did not reveal significant differences among parametric predictors for DMPFC activity, $F(2,162)=0.496$,

p=.610. Additionally, no significant differences emerged when considering linear changes in DMPFC activity as a function of parametric predictors (all *p*>.335).

NAcc. When controlling for post-scan familiarity, we observed similar findings in NAcc as when we did not control for post-scan familiarity ratings. The results did not reveal significant differences among parametric predictors for left NAcc activity, $F(2,162)=2.803$, $p=.064$. Nonetheless, because we were particularly interested in the impact of the use and availability of person-knowledge, we conducted a linear regression on parameter estimates comparing all parametric predictors. We observed a significant linear relationship between person-knowledge use and availability (contrast codes: parametric predictor for actors' body of work = .5, parametric predictor for actors' attractiveness = -.5, and parametric predictor for models' attractiveness = 0), such that left NAcc activity was greater as ratings of likability based on body of work for actors became more positive compared to when ratings of attractiveness for actors became more positive, $b=.227$, $SE=.096$, $CI_{95\%}=[.039, .416]$, $F(1,163)=5.570$, $p=.019$[i]. No other significant differences emerged: (1) ratings of likability based on body of work for actors compared to ratings of attractiveness for models, $b=.136$, $SE=.097$, $CI_{95\%}=[-.055, .327]$, $F(1,163)=1.940$, $p=.166$; and (2) ratings of attractiveness for models compared to ratings of attractiveness for actors, $b=.092$, $SE=.098$, $CI_{95\%}=[-.100, .283]$, $F(1,163)=0.881$, $p=.349$.

When controlling for post-scan familiarity ratings in the right NAcc, the results did not reveal significant differences among parametric predictors, $F(2,162)=0.111$, $p=.895$. No significant differences emerged when considering a linear change in NAcc activity as a function of parametric condition (all $p>.679$). Taken together, these results indicate that left NAcc may be sensitive to positive evaluations based on available person-knowledge relative to the mere presence of person-knowledge.

STS. When controlling for post-scan familiarity, we observed similar findings in STS as when we did not control for post-scan familiarity ratings. The results did not reveal significant differences among parametric conditions for left STS activity, $F(2,162)=0.681$, $p=.508$ or right STS activity $F(2,162)=0.986$, $p=.375$. Additionally, no significant differences emerged when considering linear changes in either left or right STS activity as a function of parametric condition (all $p>.191$).

TPJ. When controlling for post-scan familiarity, we observed similar findings in TPJ as when we did not control for post-scan familiarity ratings. The results did not reveal significant differences among parametric predictors for left, $F(2,162)=0.355$, $p=.702$, or right, $F(2,162)=0.121$, $p=.886$, TPJ activity. Additionally, no significant differences emerged when considering linear changes in either left or right TPJ activity as a function of parametric predictors (all $p>.502$).

Precuneus. When controlling for post-scan familiarity, we observed similar findings in precuneus as when we did not control for post-scan familiarity ratings. The results did not reveal significant differences among parametric predictors for precuneus activity, $F(2,162)=1.379$, $p=.255$. Additionally, no significant differences emerged when considering linear changes in precuneus activity as a function of parametric predictors (all $p>.102$).

Amygdala. When controlling for post-scan familiarity, we observed slightly different findings in the amygdala as when we did not control for post-scan familiarity ratings. The results did not reveal significant differences among parametric predictors for left amygdala activity, $F(2,162)=1.287$, $p=.279$. Additionally, no significant differences emerged when considering linear changes in left amygdala activity as a function of parametric predictors (all $p>.137$).

When controlling for post-scan familiarity in the right amygdala, the results did not reveal significant differences among parametric predictors, $F(2,162)=2.282$, $p=.105$.

Nonetheless, because we were particularly interested in the impact of the use and availability of person-knowledge, we conducted linear regressions on parameter estimates comparing all parametric predictors. As in the VMPFC, we observed a significant linear relationship between positive ratings and the combined availability/use of person-knowledge, such that right amygdala activity was greater as ratings of likability based on body of work for actors became more positive compared to when ratings of attractiveness for models became more positive, b=.393, SE=.184, $CI_{95\%}$=[.032, .754], $F(1,163)$=4.557, p=.034[i]. No other significant differences emerged: (1) ratings of likability based on body of work for actors compared to ratings of attractiveness for actors, b=.167, SE=.186, $CI_{95\%}$=[-.198, .532], $F(1,163)$=0.802, p=.372; and (2) ratings of attractiveness for actors compared to ratings of attractiveness for models , b=.226, SE=.186, $CI_{95\%}$=[-.138, .590], $F(1,163)$=1.4826, p=.225. Taken together, these results indicate that the right amygdala, like the VMPFC, may be sensitive to the use of available person-knowledge relative to the absence of person-knowledge.

Exploratory whole-brain analyses. Finally, in order to account for individual ratings of familiarity in the whole-brain exploratory analyses, we also re-analyzed these data including post-scan familiarity ratings as an additional parametric modulator in the level-1 GLM for each whole-brain parametric analysis that was reported in the main text. Separate whole-brain analyses at the second level were performed to examine changes in neural activity as a function of in-scanner ratings provided during each of the three conditions: (1) actors rated on body of work, (2) actors rated on attractiveness, and (3) models rated on attractiveness. Using the Monte Carlo simulations included in AlphaSim, the minimum cluster size required for a whole-brain correction at p<.05 with an uncorrected threshold of p<.001 was estimated to be 51 contiguous voxels.

Separate parametric analyses predicted increases in brain activity across all voxels as participants' ratings increased or decreased for each of the three conditions. Results from the whole brain analyses were also generally consistent with those presented in the main text. We summarize results from each of the whole-brain analyses below. The results for all analyses are reported in Table S1.

Impact of person-knowledge use (actors rated on body of work only). We observed greater activity in the calcarine sulcus and VMPFC as body-of-work ratings increased for the actors, but no reliable changes as body-of-work ratings decreased (Figure S1). These findings converge with the ROI findings reported above showing that VMPFC activity was sensitive to increasing positivity in body-of-work ratings for well-known actors.

Impact of person-knowledge availability (actors rated on attractiveness only). We observed greater activity in the lingual gyrus as attractiveness ratings increased for the actors, but no reliable changes as attractiveness ratings decreased.

Impact of percept-based evaluations without person-knowledge (models rated on attractiveness only). No reliable changes were observed as attractiveness ratings increased or decreased.

Table S1. Identification of BOLD signal as a function of Rating Dimension and Person-Knowledge.

Brain Region	k	*t*	*p*	MNI Coordinates		
				x	*y*	*z*
Increasing with increasing body-of-work likability ratings for familiar targets (i.e., actors)						
Calcarine Sulcus	92	4.99	< .001	-3	-60	12
VMPFC	255	4.84	< .001	6	63	0
Increasing with decreasing body-of-work likability ratings for familiar targets (i.e., actors)						
N/A						

Increasing with increasing attractiveness ratings for familiar targets (i.e., actors)							
R	Lingual Gyrus	85	4.09	< .001	36	-75	6
Increasing with decreasing attractiveness ratings for familiar targets (i.e., actors)							
	N/A						
Increasing with increasing attractiveness ratings for unfamiliar targets (i.e., models)							
	N/A						
Increasing with decreasing attractiveness ratings for unfamiliar targets (i.e., models)							
	N/A						

Note. Exploratory whole-brain analysis of 55 participants (threshold = $p < .001$, uncorrected; clusters ≥ 51 voxels determined by AlphaSim; actual values are reported in the table). Contrasts that do not identify suprathreshold regions are not reported.

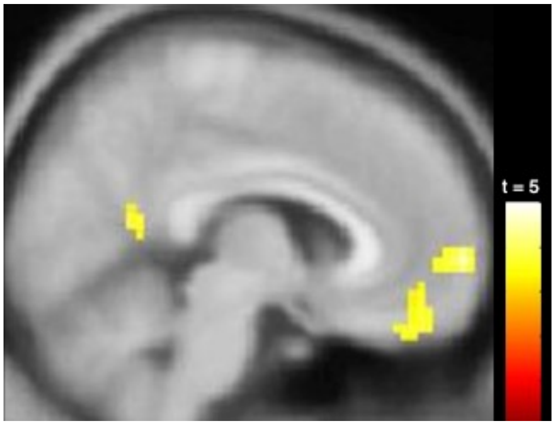

Figure S1. Brain regions associated with increases in body-of-work likability ratings for actors. The results of this exploratory whole-brain analysis are displayed on a sagittal section, x = 6 mm. Increased body-of-work ratings for the actors were associated with increasing activity in the VMPFC ($MNI_{x, y, z}$ = [6, 63, 0]) (see also calcarine sulcus).

S3: Supplementary Measures

Individual differences were assessed for an unrelated studies on race and social status[1,2] and other unrelated exploratory analyses of resting-state data.

Online Pre-Testing Part 1

Objective SES.[4]

Subjective SES.[14]

Implicit Association Test.[15]

Symbolic racism.[16]

Motivation to respond without prejudice.[17]

Current racial contact.[18]

Childhood experience questionnaire.[13]

Social dominance orientation.[19]

Intergroup anxiety.[20]

Feeling thermometers. Participants completed feeling thermometers separately for four groups of interest: high-status Black, low-status Black, high-status White, and low-status White.

Threat stereotype endorsement. Participants indicated their degree of explicit endorsement for threat stereotypes separately for four groups of interest: high-status Black, low-status Black, high-status White, and low-status White.

Social-cognitive measures for resting-state and anatomical analyses. We also assessed a number of more general social-cognitive measures for large-scale analyses of resting state and structural imaging data.

Miscellaneous demographics. Participants completed a standard battery of demographic items regarding age, sex, race/ethnicity, political affiliation, conservatism/liberalism, high school type, native language, citizenship, country of origin, and religious affiliation. Some measures of

subjective SES (e.g., family social class) and objective SES (e.g., education level and occupational prestige, childhood postal codes, K–12 educational institutions, parental education, income and occupational prestige) were also included. Participants also completed a measure of identification with their ethnic group[21].

Online Pre-Testing Part 2

Participants received links to actor online profiles and to a survey to familiarize themselves with the actors used in the fMRI experiment. Participants were instructed to study these actors prior to completing the online survey where participants had to correctly choose (50% chance of guessing) a movie in which the actor appeared or write in a different movie casting that actor.

Pre-Scan Measures

State-trait anxiety inventory.[22]

Beck Depression Inventory II.[23]

Single-item stress/anxiety scales. Participants responded to three questions about their current anxiety level, current stress level, and their stress level over the past month.

Post-Scan Measures

Social distance task. This task assessed the degree to which participants would place themselves in proximity to targets varying in race and status. At the start of the task, participants are re-introduced to four individuals pulled randomly from each of the four Race × Status conditions from the race–status fMRI experiment and paired with a name (see Supplementary Material S1). Participants were prompted to place themselves by mouse click anywhere inside of a blank white field. After clicking, the word “YOU” appeared in the clicked location. Participants were then asked if they wished to adjust the position of the word “YOU”. After any

desired adjustments for the self, participants were prompted to place each of the four faces in the same field. All previous placements remained visible. Participants were given the opportunity to adjust each placement. After all five identities were placed, participants then had one final opportunity to adjust each one in turn before finishing the task. Participants completed this task three times.

Likeability rating task. Participants completed a measure of explicit likeability for each of the 60 face stimuli presented with the same status-associated colored backgrounds used in the scanner from the race–status fMRI experiment.

Status recall. Participants indicated to the best of their ability whether each of the 60 faces from the likability task, without any status-associated color backgrounds, was low or high in status.

Trust game. We adapted a trust game[24].

Actor-model familiarity questionnaire. Participants indicated their familiarity with each actor and model used in the actor-model fMRI experiment (see main text for details).

Basic emotion states. Participants rated the degree to which they felt angry, happy, anxious, fearful, pain, and sad.

PANAS.[25]

Perceived stress.[26]

Chronic stress.[27]

Social value orientation.[28]

References

1. Mattan, B. D., Kubota, J. T., Dang, T. P. & Cloutier, J. External motivation to avoid prejudice alters neural responses to targets varying in race and status. *Soc. Cogn. Affect. Neurosci.* **13,** 22–31 (2018).
2. Mattan, B. D., Kubota, J. T., Li, T., Dang, T. P. & Cloutier, J. Motivation modulates brain networks in response to faces varying in race and status: A multivariate approach. *eNeuro* **5,** 1–13 (2018).
3. Willenbockel, V. *et al.* Controlling low-level image properties: The SHINE toolbox. *Behav. Res. Methods* **42,** 671–684 (2010).
4. Cloutier, J., Norman, G. J., Li, T. & Berntson, G. G. Person perception and autonomic nervous system response: The costs and benefits of possessing a high social status. *Biol. Psychol.* **92,** 301–305 (2013).
5. Cloutier, J., Kelley, W. M. & Heatherton, T. F. The influence of perceptual and knowledge-based familiarity on the neural substrates of face perception. *Soc. Neurosci.* **6,** 63–75 (2011).
6. Spreng, R. N., Mar, R. A. & Kim, A. S. N. The common neural basis of autobiographical memory, prospection, navigation, theory of mind, and the default mode: A quantitative meta-analysis. *J. Cogn. Neurosci.* **21,** 489–510 (2009).
7. Aharon, I. *et al.* Beautiful faces have variable reward value: fMRI and behavioral evidence. *Neuron* **32,** 537–551 (2001).
8. Kampe, K. K. W., Frith, C. D., Dolan, R. J. & Frith, U. Reward value of attractiveness and gaze. *Nature* **413,** 589–602 (2001).
9. Cloutier, J., Heatherton, T. F., Whalen, P. J. & Kelley, W. M. Are attractive people rewarding? Sex differences in the neural substrates of facial attractiveness. *J. Cogn.*

Neurosci. **20,** 941–951 (2008).

10. Deen, B., Koldewyn, K., Kanwisher, N. & Saxe, R. Functional organization of social perception and cognition in the superior temporal sulcus. *Cereb. Cortex* **25,** 4596–4609 (2015).

11. Saxe, R. Uniquely human social cognition. *Curr. Opin. Neurobiol.* **16,** 235–239 (2006).

12. Adolphs, R. & Spezio, M. Role of the amygdala in processing visual social stimuli. *Prog. Brain Res.* **156,** 363–378 (2006).

13. Cloutier, J., Li, T. & Correll, J. The impact of childhood experience on amygdala response to perceptually familiar Black and White faces. *J. Cogn. Neurosci.* **26,** 1992–2004 (2014).

14. Adler, N. E., Epel, E. S., Castellazzo, G. & Ickovics, J. R. Relationship of subjective and objective social status with psychological and physiological functioning: Preliminary data in healthy white women. *Heal. Psychol.* **19,** 586–592 (2000).

15. Greenwald, A. G., McGhee, D. E. & Schwartz, J. L. Measuring individual differences in implicit cognition: The implicit association test. *J. Pers. Soc. Psychol.* **74,** 1464–1480 (1998).

16. Kinder, D. R. & Sears, D. O. Prejudice and politics: Symbolic racism versus racial threats to the good life. *J. Pers. Soc. Psychol.* **40,** 414–431 (1981).

17. Plant, E. A. & Devine, P. G. Internal and external motivation to respond without prejudice. *J. Pers. Soc. Psychol.* **75,** 811–832 (1998).

18. Pettigrew, T. F. Generalized intergroup contact effects on prejudice. *Personal. Soc. Psychol. Bull.* **23,** 173–185 (1997).

19. Pratto, F., Sidanius, J., Stallworth, L. M. & Malle, B. F. Social dominance orientation: A personality variable predicting social and political attitudes. *J. Pers. Soc. Psychol.* **67,** 741–763 (1994).

20. Greenland, K., Xenias, D. & Maio, G. Intergroup anxiety from the self and other: Evidence from self-report, physiological effects, and real interactions. *Eur. J. Soc. Psychol.* **42,** 150–163 (2012).

21. Sidanius, J., Feshbach, S., Levin, S. & Pratto, F. The interface between ethnic and national attachment: Ethnic pluralism or ethnic dominance? *Public Opin. Q.* **61,** 102–133 (1997).

22. Spielberger, C. D., Gorsuch, R. L., Lushene, R., Vagg, P. R. & Jacobs, G. A. *Manual for the State-Trait Anxiety Inventory*. (Consulting Psychologists Press, 1983).

23. Beck, A. T., Steer, R. A. & Brown, G. K. *Manual for the Beck Depression Inventory-II.* (Psychological Corporation, 1996).

24. Berg, J., Dickhaut, J. & McCabe, K. Trust, reciprocity, and social history. *Games Econ. Behav.* **10,** 122–142 (1995).

25. Watson, D., Clark, L. A. & Tellegen, A. Development and validation of brief measures of positive and negative affect: The PANAS scales. *J. Pers. Soc. Psychol.* **54,** 1063–1070 (1988).

26. Cohen, S. & Williamson, G. Perceived stress in a probability sample of the United States. in *The Social Psychology of Health. The Claremont Symposium on Applied Social Psychology* (eds. Spacapan, S. & Oskamp, S.) 31–67 (Sage, 1988).

27. Turner, R. J., Wheaton, B. & Lloyd, D. A. The epidemiology of social stress. *Am. Sociol. Rev.* **60,** 104–125 (1995).

28. Van Lange, P. A., Otten, W., De Bruin, E. M. & Joireman, J. A. Development of prosocial, individualistic, and competitive orientations: Theory and preliminary evidence. *J. Pers. Soc. Psychol.* **73,** 733–746 (1997).
